# Supplementary material for: Angiopoietin-2 and the Vascular Endothelial Growth Factor Promote Migration and Invasion in Hepatocellular Carcinoma- and Intrahepatic Cholangiocarcinoma-Derived Spheroids
Source: Biomedicines. 2023 Dec 30;12(1):87. doi: 10.3390/biomedicines12010087 (PMC10813100; doi:10.3390/biomedicines12010087)
Supplement: Supplementary file 1 [file biomedicines-12-00087-s001.zip › biomedicines-2778950-supplementary-updated.pdf]

Supplementary materials and methods

Cell cultures

HepG2 were grown in Eagle's MEM (Merck Life Science S.r.l., Milan, Italy) supplemented with 10% of FBS, 1% of non-essential amino acids solution, 1% of glutamine and 1% of Penicillin-Streptomycin. Hep3B cells were grown as HepG2 with the addition of 1% of sodium pyruvate (all purchased from Gibco, Thermo Fisher Scientific, Waltham, MA, USA). HuCCT-1 cells and EGI-1 cells were grown in RPMI 1640 (Merck Life Science S.r.l., Milan, Italy) supplemented with 10% of FBS, 1% of glutamine and 1% of Penicillin-Streptomycin.

Gene expression analysis

RNA from spheroids was extracted with Nucleozol (Mechery-Nagel GmbH & Co. KG, Dueren, Germany) and purified with NucleoSpin columns (Mechery-Nagel GmbH & Co. KG). After quantification and cDNA synthesis, a mix of reactions for digital PCR containing ddPCR master mix (Bio-Rad, Segrate, Italy), primers for each target (Thermo Fisher Scientific, Waltham, MA, USA) and sample nucleic acid solution was prepared. The droplets were prepared with Automated Droplet Generator (Bio-Rad Laboratories, Segrate, Italy) and transferred in 96 well PCR plates. PCR amplification was carried out on C-1000 Touch Thermal cycler (Bio-Rad Laboratories, Segrate, Italy). After amplification, the droplets for each well were read automatically with QX200 Droplet Reader (Bio-Rad Laboratories, Segrate, Italy). All procedures were performed according to the manufacturer's instructions.

Western blot analysis

Spheroids were lysed in a cold RIPA buffer supplemented with a protease and phosphatase inhibitors cocktail (Roche Applied Science, Mannheim, Germany). Proteins were quantified using a BCA method (Quantum micro protein kit, Euroclone Spa, Milan, Italy), were run on 8-10% polyacrylamide gels and electrotransferred to nitrocellulose membrane (Bio-Rad Laboratories, Segrate, Italy). After blocking, membranes were probed with primary antibodies overnight at +4 °C and incubated with specific secondary antibodies (R&D Systems, Milan, Italy) for 1 h RT. Protein bands were visualized by enhanced chemiluminescent substrate (Cyanagen srl, Bologna, Italy) using Chemidoc MP (Bio-Rad Laboratories, Segrate, Italy). The following antibodies were used: E-cadherin (BD Biosciences, Franklin Lakes, NJ, USA), N-cadherin (Thermo Fisher Scientific, Waltham, MA, USA), Vimentin (BD Biosciences), ANG-2 (R&D Systems), VEGFA (Cell Signaling Technology, Danvers, MA, USA), TIE2 (Santa Cruz Biotechnology, Delaware Ave, Santa Cruz, CA), phopsho-TIE2 (R&D Systems, Milan, Italy), VEGFR1 (Santa Cruz Biotechnology, Delaware Ave, Santa Cruz, CA), phospho VEGFR1 (R&D Systems, Milan, Italy) and β-actin (R&D Systems, Milan, Italy). To evaluate the expression of phosphorylated proteins, the membranes probed with antibodies anti-TIE2 or -VEGFR1, were stripped and reprobed with antibodies against P-TIE2 or P-VEGFR1. All protein bands were quantified by densitometry using ImageLab software (Bio-Rad Laboratories, Segrate, Italy) and normalized to β-actin content.

Immunofluorescence analysis

Spheroids fixed in formaldehyde, were blocked with Image IT Fix (Invitrogen, Waltham, MA, USA), and incubated with antibodies directed to E-cadherin (BD Biosciences, Franklin Lakes, NJ, USA) and N-cadherin (Thermo Fisher Scientific, Waltham, MA, USA). Then, spheroids were incubated with fluorochrome-conjugated secondary antibodies (Alexa Fluor 488 and Alexa Fluor 546, Life Technologies). Nuclei were stained with DAPI (Thermo Fisher Scientific, Waltham, MA, USA).

Supplementary data

Analysis of ANG-2 and VEGFA protein expressions.

We detected ANG-2 and VEGFA expression in HepG2 and HuCC-T1, and Hep3B spheroids, respectively, at time intervals of 15, 30 and 60 min following stimulation, as shown in Figure S2. Our ANG-2 analysis showed two distinct bands in the samples stimulated with rh-ANG-2. The band with the higher molecular weight likely represents the added, highly glycosylated recombinant protein. In contrast, control and rh-VEGF-stimulated samples exhibited only a single band. This might represent the endogenously produced protein. (Figure S2 A-D). In both cell lines ANG-2 detected was significantly higher in treated cells than in untreated with rh-ANG-2. The expression levels of VEGFA in Hep3B spheroids was significantly higher over time in rh-VEGF treatments (both alone or combined with rh-ANG-2) than untreated or treated with rh-ANG-2 alone (Figure S2 E, F).

EMT marker gene expression

To ascertain the impact of rh-ANG-2 and/or rh-VEGF stimulation on EMT marker expression, we conducted gene analyses for E-cadherin (CDH1), N-cadherin (CDH2), and Vimentin (VIM) in lysates derived from spheroids. Transcriptomic analyses revealed the expression of EMT markers across all four cell lines. Specifically, an upregulation of CDH2 was noted in HepG2 cells, while an increase in VIM expression was observed in both HCC and iCCA cell lines. A decline in CDH1 expression was identified uniquely in Hep3B cells, although changes were sporadically treatment-dependent (Figure S3). EGI-1 cells exhibited low expression levels of CDH2 and VIM genes.

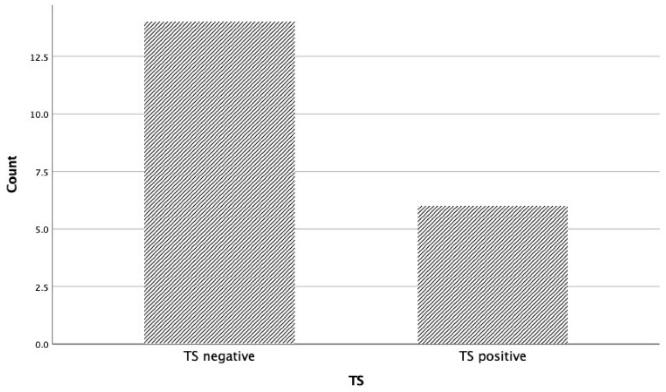

Figure S1. We evaluated the expression of 5-gene 'neoangiogenic' transcriptomic signature (TS) in 20 biopsies of iCCA. We have found that 6 (20%) were positive, while 14 (80%) were negative for TS.

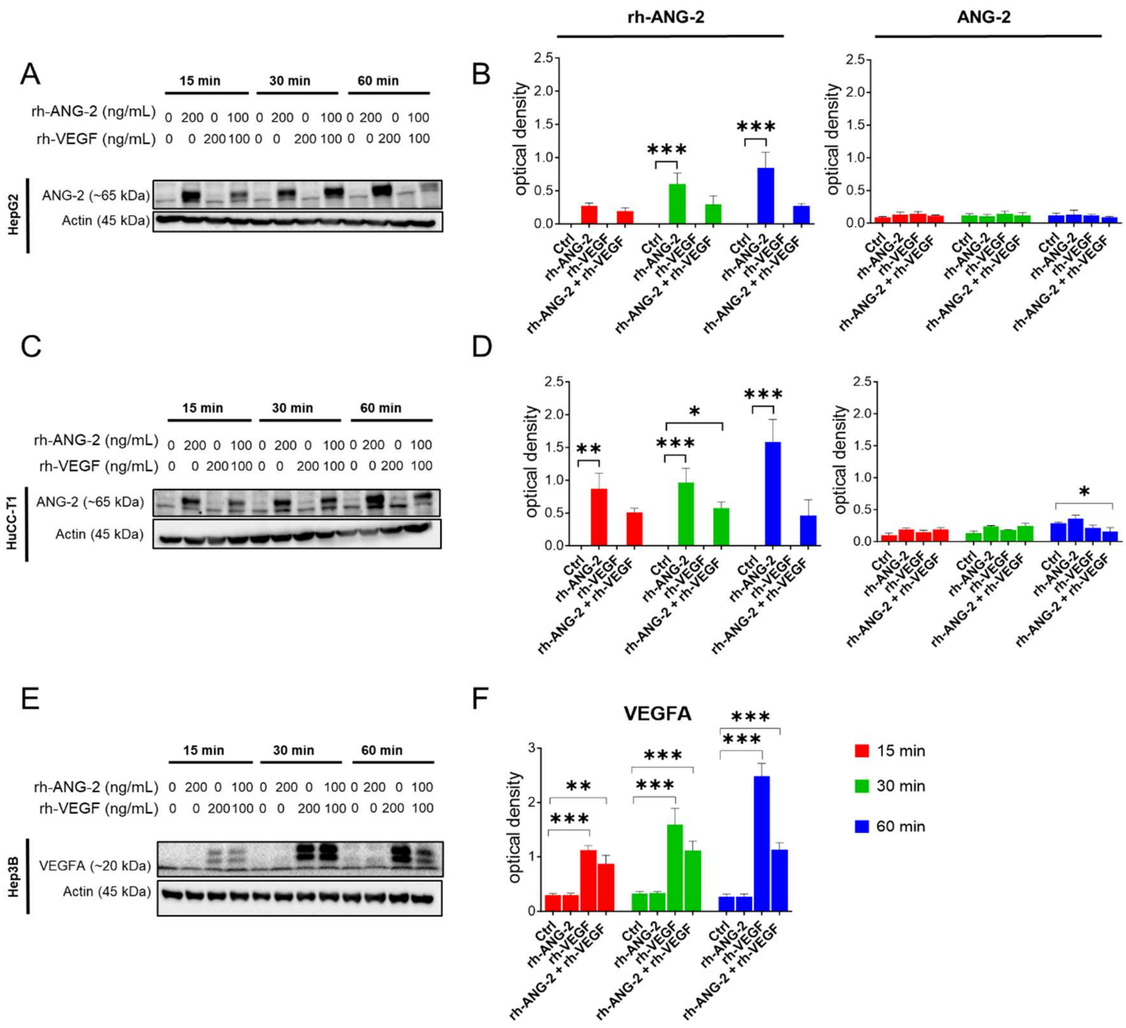

Figure S2. We evaluated the expression of ANG-2 in HepG2 and HuCC-T1 spheroids (A-D), and the expression of VEGFA in Hep3B spheroids (E,F). Treatments included 200 ng/mL of rh-ANG-2, 200 ng/mL of rh-VEGF, and a combination of 100 ng/mL each of rh-ANG-2 and rh-VEGF. Proteins were harvested at 15, 30 and 60 min post-stimulation. Band intensities were quantified using ImageLab software, and densitometry data were presented as optical density values normalized to

β-actin. Comparative analyses were performed between the treated spheroids and controls at identical time points. This experiment was replicated a minimum of three times. Statistical differences were assessed using two-way ANOVA, with subsequent post-hoc tests for multiple comparisons. Significance thresholds were established as: *$p < 0.05$; ** $p < 0.01$; *** $p < 0.001$.

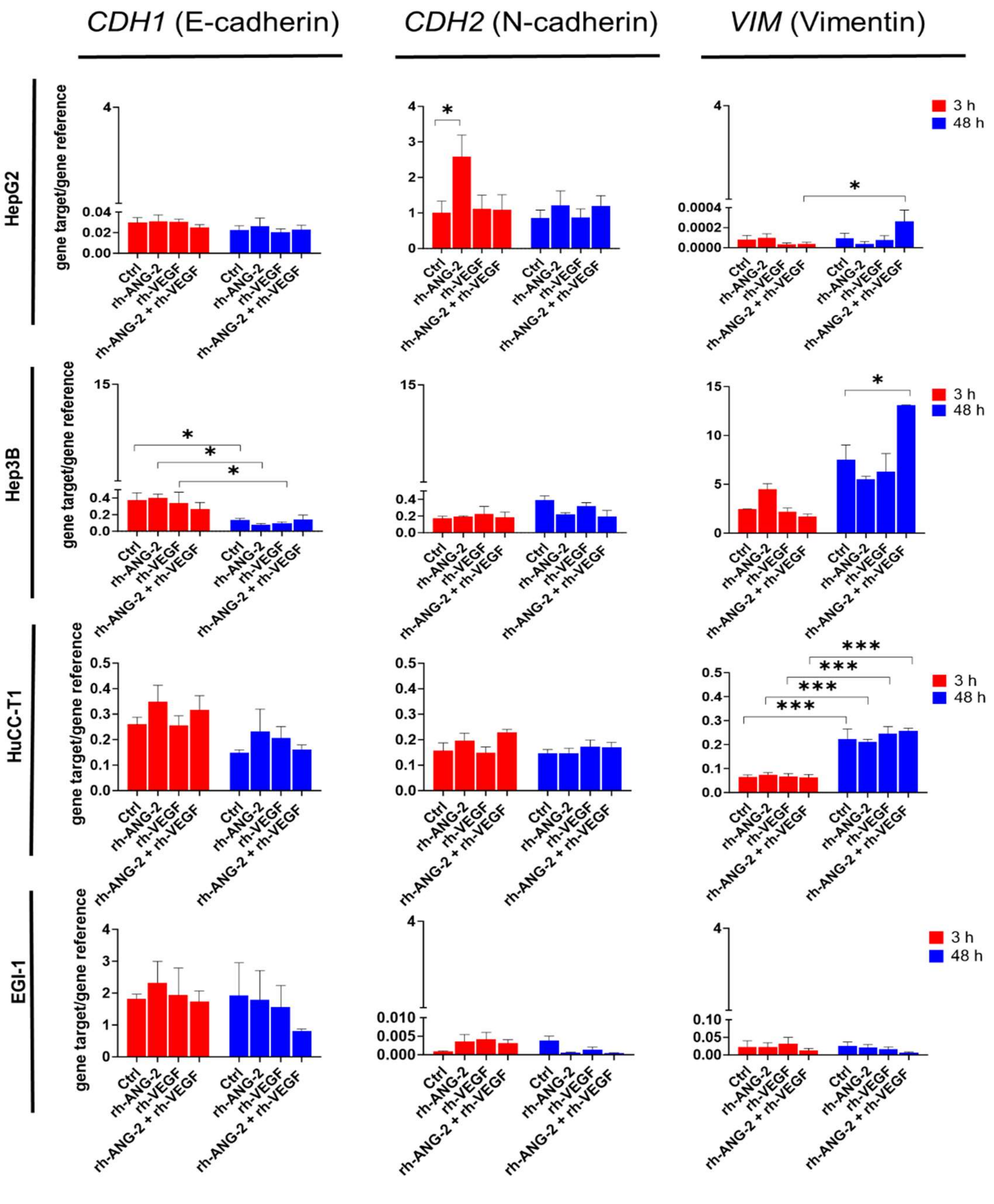

Figure S3. ddPCR analysis of EMT markers gene expression. We assessed the gene expression levels of the EMT markers CDH1, CDH2, and VIM in spheroids derived from HepG2, Hep3B, HuCC-T1, and EGI-1. These spheroids were either untreated (Ctrl) or subjected to treatment with 200 ng/mL of rh-ANG-2 alone, 200 ng/mL of rh-VEGF alone, or a combination of 100 ng/mL each of rh-ANG-2 and rh-VEGF. RNA was extracted at 3 and 48 h post-treatment, and the number of gene target copies was normalized to reference gene copies. Comparative analyses were conducted between the treated spheroids and controls at the corresponding time points, as well as between the spheroid gene expression levels at 48 h and 3 h within the same treatment condition. These assays were performed in triplicate to ensure data reliability.

Statistical differences between groups were evaluated using two-way ANOVA, complemented by post-hoc tests for multiple comparisons. Significance levels are established as: * $p < 0.05$; *** $p < 0.001$.

Table S1: Summary of western blot analysis for EMT markers in HepG2, Hep3B, HuCC-T1 and EGI-1 spheroids.

		3 h			48 h		
		E-cadherin	N-cadherin	Vimentin	E-cadherin	N-cadherin	Vimentin
**HepG2**	Ctrl				↓ vs. ctrl 3h	↑	↑
	A	↓	↑	=	↓	↑	↑
	V	↓	↑	=	↓	↑	↑ vs. V 3h vs. ctrl 48h
	A+V	↓	↑	=	↓	↑	↑ vs. A+V 3h vs. ctrl 48h
**Hep3B**	Ctrl				↓	↑	=
	A	=	=	=	↓	↑	↑
	V	=	=	=	↓	↑ vs. V 3h	↑ vs. V 3h
	A+V	↑	=	=	↓ vs. A+V 3h	↑ vs. A+V 3h	↑ vs. A+V 3h
**HuCC-T1**	Ctrl				=	=	=
	A	=	=	=	=	=	↑ vs. A+V 3h
	V	=	=	=	=	=	=
	A+V	=	=	=	=	=	=
**EGI-1**	Ctrl						
	A	=			=		
	V	=			=		
	A+V	=			=		

The colored arrows (red for down regulation and green for up regulation) indicate significant differences. Black arrows indicate non-significant differences. Where not specified, at 3 h, the colored arrows refer to comparison between treatment (rh-Ang-2 [A], rh-VEGF [V], rh-ANG-2 plus rh-VEGF [A+V]) and control (Ctrl).
